# 5-Aminolevulinic Acid Improves Nutrient Uptake and Endogenous Hormone Accumulation, Enhancing Low-Temperature Stress Tolerance in Cucumbers

**DOI:** 10.3390/ijms19113379

**Published:** 2018-10-29

**Authors:** Ali Anwar, Yan Yan, Yumei Liu, Yansu Li, Xianchang Yu

**Affiliations:** 1 The Institute of Vegetables and Flowers, Chinese Academy of Agricultural Sciences, Beijing 100081, China; dr.ali_ivf@yahoo.com (A.A.); yanyan101@163.com (Y.Y.); sd.liuyumei@163.com (Y.L.); 2 College of Agricultural and Biological Engineering, Heze University, Heze 274015, China

**Keywords:** ALA, cucumber, antioxidant enzymes, hormones, nutrient, low temperature

## Abstract

5-aminolevulinic acid (ALA) increases plant tolerance to low-temperature stress, but the physiological and biochemical mechanisms that underlie its effects are not fully understood. To investigate them, cucumber seedlings were treated with different ALA concentrations (0, 15, 30 and 45 mg/L ALA) and subjected to low temperatures (12/8 °C day/night temperature). The another group (RT; regular temperature) was exposed to normal temperature (28/18 °C day/night temperature). Low-temperature stress decreased plant height, root length, leaf area, dry mass accumulation and the strong seedling index (SSI), chlorophyll contents, photosynthesis, leaf and root nutrient contents, antioxidant enzymatic activities, and hormone accumulation. Exogenous ALA application significantly alleviated the inhibition of seedling growth and increased plant height, root length, hypocotyl diameter, leaf area, and dry mass accumulation under low-temperature stress. Moreover, ALA increased chlorophyll content (Chl a, Chl b, Chl a+b, and Carotenoids) and photosynthetic capacity, net photosynthetic rate (*Pn*), stomatal conductance (*Gs*), intercellular CO_2_ concentration (*Ci*), and transpiration rate (*Tr*), as well as the activities of superoxide dismutase (SOD), peroxidase (POD, catalase (CAT), ascorbate peroxidase (APX), and glutathione reductase (GR) enzymes, while decreasing hydrogen peroxide (H_2_O_2_), superoxide (O_2_^•−^), and malondialdehyde (MDA) contents under low-temperature stress. In addition, nutrient contents (N, P, K, Mg, Ca, Cu, Fe, Mn, and Zn) and endogenous hormones (JA, IAA, BR, iPA, and ZR) were enhanced in roots and leaves, and GA4 and ABA were decreased. Our results suggest the up-regulation of antioxidant enzyme activities, nutrient contents, and hormone accumulation with the application of ALA increases tolerance to low-temperature stress, leading to improved cucumber seedling performance.

## 1. Introduction

Plants are challenged by a variety of biotic and abiotic stresses throughout their life cycle [1]. Low-temperature stress limits agriculture production severely in large parts of the world, especially northern parts of China [2,3,4]. Low temperature is one of the most common problems for offseason vegetable production [2,3]. Numerous studies have shown that low temperatures negatively affect plant nutritional uptake and accumulation, chlorophyll content, photosynthetic capacity, oxidative stress, metabolic processes, defense system, and hormonal imbalance [5], in addition to having adverse effects on almost all developmental stages from seed germination to maturation [6]. Plants exposed to low temperatures can increase the overproduction of ^1^O_2_, O_2_^•−^, H_2_O_2_, and OH, also known as reactive oxygen species (ROS), which damage chloroplasts and mitochondria and can lead to cell death [7]. To eliminate the overproduction of ROS, plants have evolved antioxidant enzymes (superoxide dismutase (SOD), peroxidase (POD), catalase (CAT), ascorbate peroxidase (APX), glutathione reductase (GR), and dehydroascorbate reductase (DHAR)) and nonenzymatic antioxidants (glutathione (GSH), ascorbic acid (AA), and carotenoids) that are responsible for scavenging superfluous ROS [6,7,8]. Phytohormones (abscisic acid (ABA), indole-3-acetic acid (IAA), gibberellin (GA), brassinosteroid (BR), jasmonic acid (JA), and indole-3-propionic acid (iPA)) also play key roles in increasing antioxidant enzyme activities under various kinds of abiotic stress to reduce their harmful effects [5,9,10,11].

5-aminolevulinic acid (ALA) is a kind of nonprotein amino acid found in plants, animals, fungi, and bacteria [8]. ALA is a key precursor in the biosynthesis of all porphyrin compounds, like chlorophyll, heme, and phytohormones [11]. Exogenous ALA application has been reported to regulate chlorophyll biosynthesis and photosynthesis, thus increasing crop yields [8,12]. As an essential biosynthetic precursor of all heterocyclic tetrapyrrole molecules, ALA is considered a plant growth regulator, and is involved in improvements in plant growth and yield, and variety of abiotic stress tolerance, suggesting that ALA has great application potential in agriculture production, because it is a nontoxic endogenous substance [8,13,14]. ALA alleviates the harmful effects of salinity as well as increasing the chlorophyll content, photosynthetic rate, antioxidant enzymatic capacity, and nutrient content [8]. Exogenous ALA application increases the plant defense system in response to NaCl [12], low-temperature stress and light condition [13], salinity stress [14], and drought [15]. In rice, microarray analysis suggests that ALA upregulated transcripts highlight particular biological processes, transcription factors, post-transcription factors, signal transduction, carbohydrate and monoacids metabolism, and chlorophyll biosynthesis [16] thus, leads to increase stress tolerance [17,18,19]. These findings suggest that ALA can broadly reduce the harmful effects of abiotic stress, but little about its role in endogenous hormone regulations in response to low-temperature stress is known.

The optimum growth temperature for cucumber is between 24 and 28 °C [9,20]. Temperatures above 30 °C or below 12 °C cause significant reduction in growth [9,10]. Subjecting cucumber plants to low-temperature stress can also induce ROS and malondialdehyde (MDA) production in leaves, and increase their susceptibility to a variety of diseases and pathogens [7,11]. Low-temperature stress reduced chlorophyll biosynthesis [2,14,17], impaired photosynthesis and respiration [21,22], membrane damage, overproduction of ROS [8], and hormonal imbalance [10,11,12,13], causing a significant reduction in plant growth and yield [23]. China is the world leader in cucumber production, in part due to intensive cultivation in the northern region, where the climate is ideal for growing cucumber. However, low temperatures in early spring are a problem for many horticulture crops, including cucumber [21].

Here, we provide the first evidence that ALA protects cucumber seedlings against low-temperature stress by regulating endogenous hormones levels. We also investigate ALA’s role in promoting low-temperature stress tolerance, and would be useful for greenhouse and protected vegetables production.

## 2. Results

### 2.1. The Effect of ALA on Cucumber Seedling Growth and Strong Seedling Index

The results of the present study indicate that low-temperature stress had a negative effect on cucumber seedling growth and the strong seedling index (SSI), but these were significantly enhanced by exogenous ALA application (Table 1). Plant height, root length, hypocotyl diameter, leaf area, plant dry weight, and SSI of cucumber seedlings were significantly reduced by 41.73%, 42.11%, 26.55%, 44.78%, 48.65%, and 36.84%, respectively, in low-temperature stress (CK), and by 14.93%, 19.14%, 5.19%, 18.93%, 24.32%, and 10.53%, respectively, in T2 (30 mg/L ALA), when compared to RT (regular temperature). ALA treatment T2 significantly enhanced plant height, root length, hypocotyl diameter, leaf area, plant dry weight, and SSI by 46%, 39.68%, 29.07%, 46.81%, 47.36%, and 41.66%, respectively, compared with CK. SSI was highest in RT followed by T2 and T3 treatments, respectively. SSI was significantly lower in CK and T1, which did not differ from one another (Table 1). These results suggest that exogenous ALA reduced the harmful effects of low-temperature and also increased cucumber seedling size metrics, as measured seven days after exposure to low-temperature stress (Table 1). Among the treatments T2 showed the greatest growth and was the only treatment selected for further analysis.

### 2.2. The Effect of ALA on Chlorophyll and Photosynthesis

**Figure 1:**
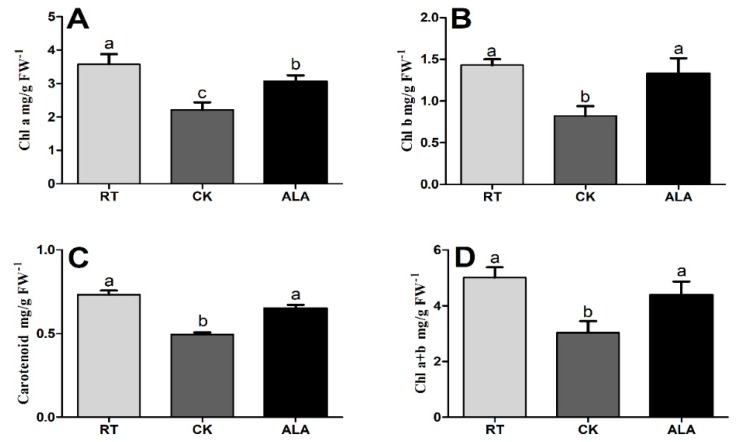
The effect of ALA on chlorophyll (**A**: Chlorophyll A (Chl a); **B**: Chlorophyll b (Chl b); **C**: Carotenoid; and **D**: Chlorophyll a+b (Chl a+b)) accumulation under low-temperature stress conditions. RT = regular temperature, CK = control; ALA = 30 mg/L ALA. Treatments indicated by the same letters are not significantly different at *p* < 0.05.

Low-temperature stress induced a significant reduction in chlorophyll contents (Chl a, Chl b, Chl a+b, and carotenoid) (Figure 1). The results showed that Chl a, Chl b, Chl a+b, and carotenoid contents in CK decreased by 38.27%, 42.46%, 32.88%, and 39.32%, respectively, and 14.25%, 7.28%, 11.32%, and 12.12% in ALA treatments, when compared to the RT treatment. The Chl b, Chl a+b, and carotenoid contents of RT were not significantly different than ALA, while Chl a was significantly higher in RT. Moreover, Chl a, Chl b, Chl a+b, and carotenoid contents decreased by 27.37%, 38.10%, 23.98%, and 30.87%, respectively, in the low-temperature stress (CK) treatment, as compared with ALA treatment. The findings suggest that ALA protected chlorophyll contents under low temperature stress.

**Figure 2:**
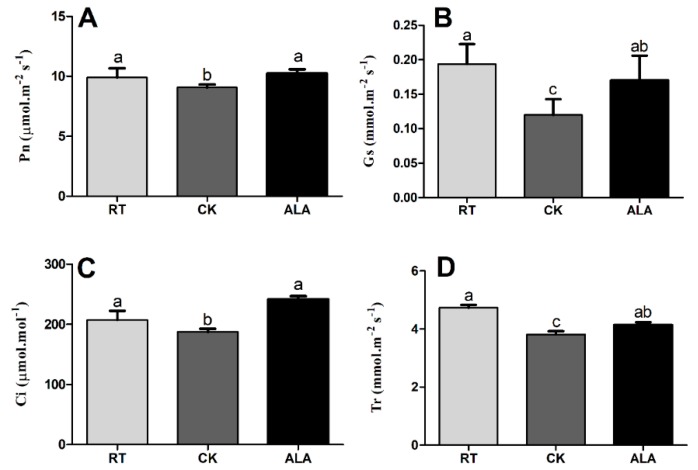
The effect of ALA on photosynthetic parameters (*Pn*; net photosynthesis (**A**), *Gs*; stomatal conductance (**B**), *Ci*; intercellular CO_2_ concentration (**C**), and *Tr*; transpiration rate (**D**)) under low-temperature stress. RT = regular temperature; CK = control; ALA = 30 mg/L ALA. Different letters indicate a significant difference at *p* < 0.05.

The photosynthetic capacity was significantly enhanced by exogenous ALA application in cucumber seedling under low temperature stress (Figure 2). The results indicate that net photosynthesis (*Pn*), stomatal conductance (*Gs*), intercellular CO_2_ concentration (*Ci*), and transpiration rate (*Tr*) decreased by 11.32%, 29.41%, 22.36%, and 7.73%, respectively, compared to ALA-treated seedlings (Figure 2). Compared to the RT treatment, the *Pn*, *Gs*, *Ci*, and *Tr* in CK decreased by 8.28%, 38.12%, 9.48%, and 19.2%, respectively. ALA treatment resulted in a remarkable increase in *Pn* and *Ci*, by 3.43% and 16.59%, but *Gs* and *Tr* decreased by 12.33% and 12.66%, respectively, suggesting that ALA reduced the damaging effects of low-temperature stress by increasing photosynthetic capacity. Together, these findings indicate that ALA increases chlorophyll contents and photosynthetic capacity in cucumber under low-temperature stress.

### 2.3. Effect of ALA on Antioxidant Enzyme Activities, MDA, and ROS Contents

**Figure 3:**
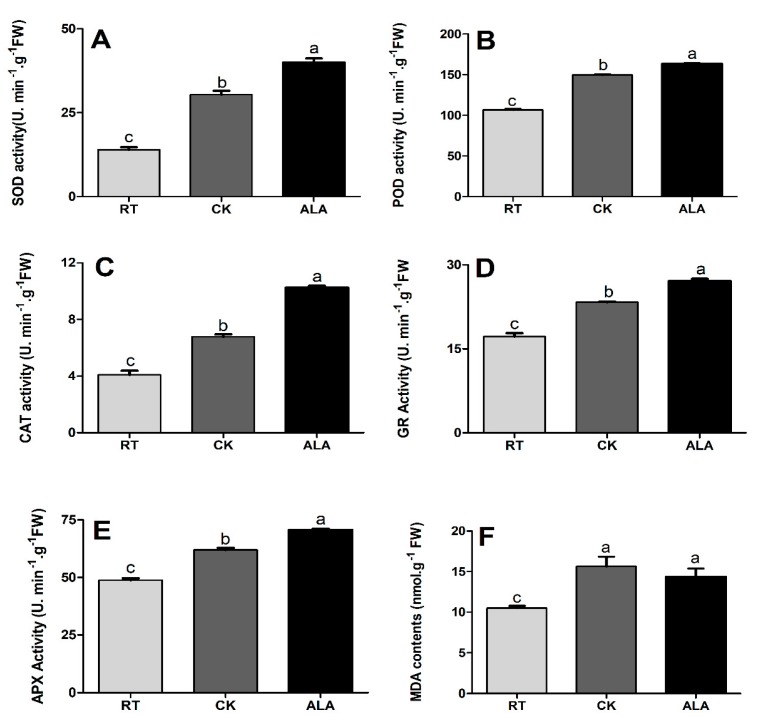
The effect of ALA on antioxidant enzymatic activities (**A**: SOD = superoxide dismutase, **B**: POD = peroxidase, **C**: CAT = catalase, **D**: APX = ascorbate peroxidase, **E**: GR = glutathione reductase, and **F**: MDA = malondialdehyde) under low-temperature stress in cucumber seedlings. RT = regular temperature; CK = control; ALA = 30 mg/L ALA. Different letters indicate a significant difference at *p* < 0.05.

Plants subjected to abiotic stress tend to overproduce reactive oxygen species (ROS), which can lead to oxidative stress that damages proteins, lipids, carbohydrates, chlorophyll, and the photosynthetic machinery. Plants have antioxidant enzymes (SOD, POD, CAT, APX, and GR) that respond to stress tolerance and regulate ROS and MDA production. Compared with the ALA treatment, the SOD, POD, CAT, APX, and GR enzymatic activities in cucumber seedling leaves were significantly reduced in CK. The results show that exogenous ALA application increased the SOD, POD, CAT, APX, and GR enzymatic activities, by 31.33%, 9.09%, 50.82%, 14.05%, and 15.99%, respectively, as compared to CK (Figure 3). Compared with RT, the SOD, POD, CAT, APX, and GR enzyme activities were enhanced by 54.15%, 28.67%, 39.72%, 36.30%, and 21.33%, respectively, in the CK treatment, and 65.10%, 34.61%, 60.03%, 57.26%, and 31.02%, respectively, in the ALA treatment (Figure 3).

**Figure 4:**
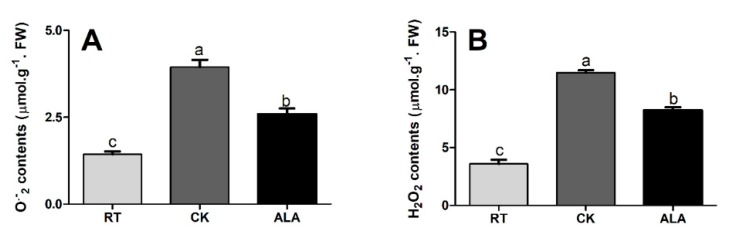
The effect of ALA on O_2_^•−^; superoxide (**A**) and H_2_O_2_; hydrogen peroxide (**B**) levels under low-temperature stress. RT = regular temperature; CK = control; ALA = 30 mg/L ALA. Different letters indicate a significant difference at *p* < 0.05.

Further, we investigated the H_2_O_2_ and O_2_^•−^ and MDA contents in cucumber seedling leaves. Low-temperature stress increased the levels of H_2_O_2_ and O_2_^•−^ and MDA, by 39.51%, 52.26%, and 12.91%, respectively, relative to seedlings treated with ALA (Figure 4). Moreover, H_2_O_2_ and O_2_^•−^ and MDA contents were significantly lower in RT, but increased by 68.60%, 63.63%, and 32.80%, respectively, in the CK treatment and 56.12%, 42.68%, and 26.91% in ALA-treated seedlings (Figure 4). These findings suggest ALA regulates a plant’s defense system to reduce ROS overproduction and improve growth under low temperature stress.

### 2.4. Effect of ALA on Total Nutrient Contents

**Figure 5:**
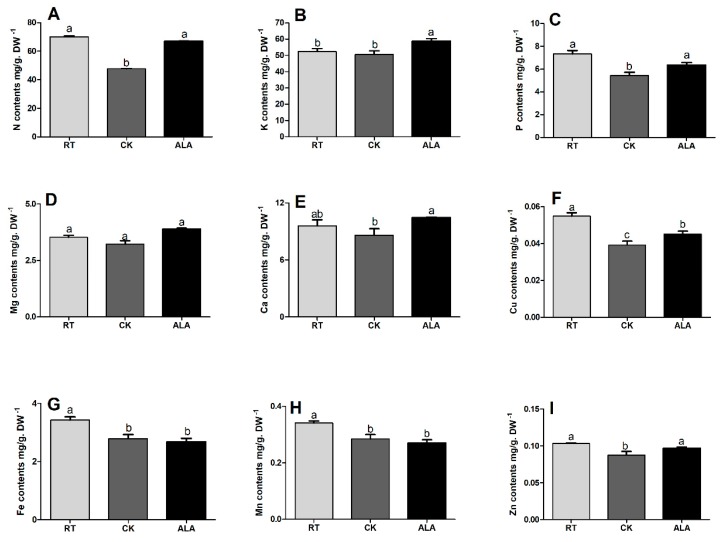
Effect of ALA on root nutrient contents (**A**; Nitrogen, **B**; Potassium, **C**; Phosphorus, **D**; Magnesium, **E**; Calcium, **F**; Copper, **G**; Iron, **H**; Manganese, and **I**; Zinc) under low-temperature stress. RT = regular temperature; CK = control; ALA = 30 mg/L ALA. Different letters indicate a significant difference at *p* < 0.05.

**Figure 6:**
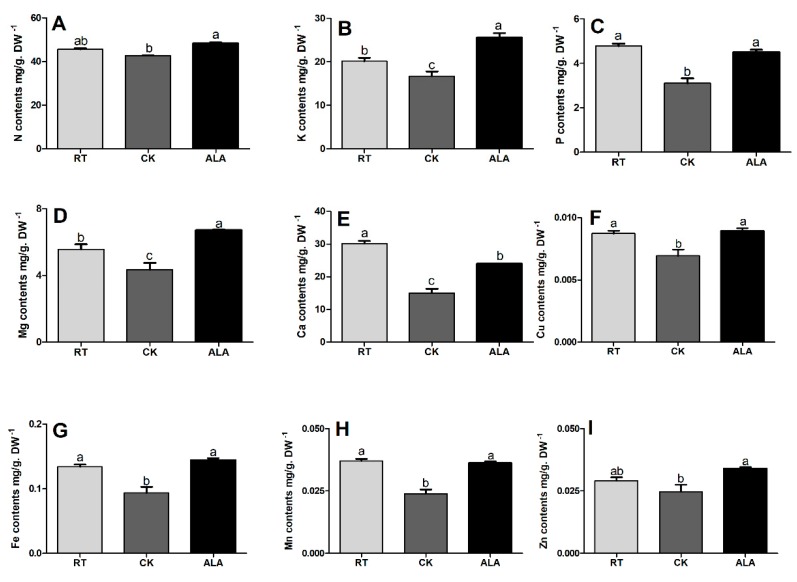
Effect of ALA on nutrient contents (**A**; Nitrogen, **B**; Potassium, **C**; Phosphorus, **D**; Magnesium, **E**; Calcium, **F**; Copper, **G**; Iron, **H**; Manganese, and **I**; Zinc) in cucumber seedling leaves under low-temperature stress. RT = regular temperature; CK = control; ALA = 30 mg/L ALA. Different letters indicate a significant difference at *p* < 0.05.

Exogenous ALA application leads to significant changes in root and leaf nutrient content when cucumber seedlings were exposed to low-temperature stress compared to the CK treatment (Figure 5 and Figure 6). The N, K, P, Mg, Cu, Ca, and Zn contents in root of ALA treated seedlings, increased by 28.94%, 14.01%, 14.66%, 17.27%, 12.62%, 17.71%, and 9.83%, respectively, but Mn and Fe contents were the same compared to CK (Figure 5). Moreover, N, K, P, Mg, Cu, Ca, and Zn contents in roots of CK seedlings decreased by 32.07%, 3.40%, 25.92%, 8.52%, 10.28%, 28.73%, 18.66%, 16.18%, and 15.53%, respectively, as compared to RT (Figure 5). The ALA treated seedlings showed a remarkable increase in K, Mg, Ca by 12.34%, 10.51% and 8.99%, respectively, but N, P, Cu, Fe, Mn, and Zn contents decreased by 4.67%, 13.23%, 18.55%, 21.87%, 20.59%, and 5.92%, respectively.

In addition, ALA also increases leaf nutrient content of N, K, P, Mg, Mn, Cu, Fe, Ca, and Zn compared to the CK treatment by 12.49%, 4.84%, 31.15%, 35.35%, 34.13%, 22.27%, 35.10%, 37.51%, and 27.17%, respectively (Figure 6). To compared with RT, the N, K, P, Mg, Mn, Cu, Fe, Ca and Zn of leaves decreased by 6.33%, 16.95%, 34.94%, 21.94%, 50.22%, 21.84%, 28.08%, 35.54%, and 15.07%, respectively, in the CK treatment, but the ALA treatment showed no significant difference with RT (Figure 6). Taken together, these results suggest that the difference between RT and ALA were not significant, but CK and RT resulted in a significant difference under low temperature stress in cucumber seedling.

### 2.5. Effect of ALA on Endogenous Hormones Accumulation

**Figure 7:**
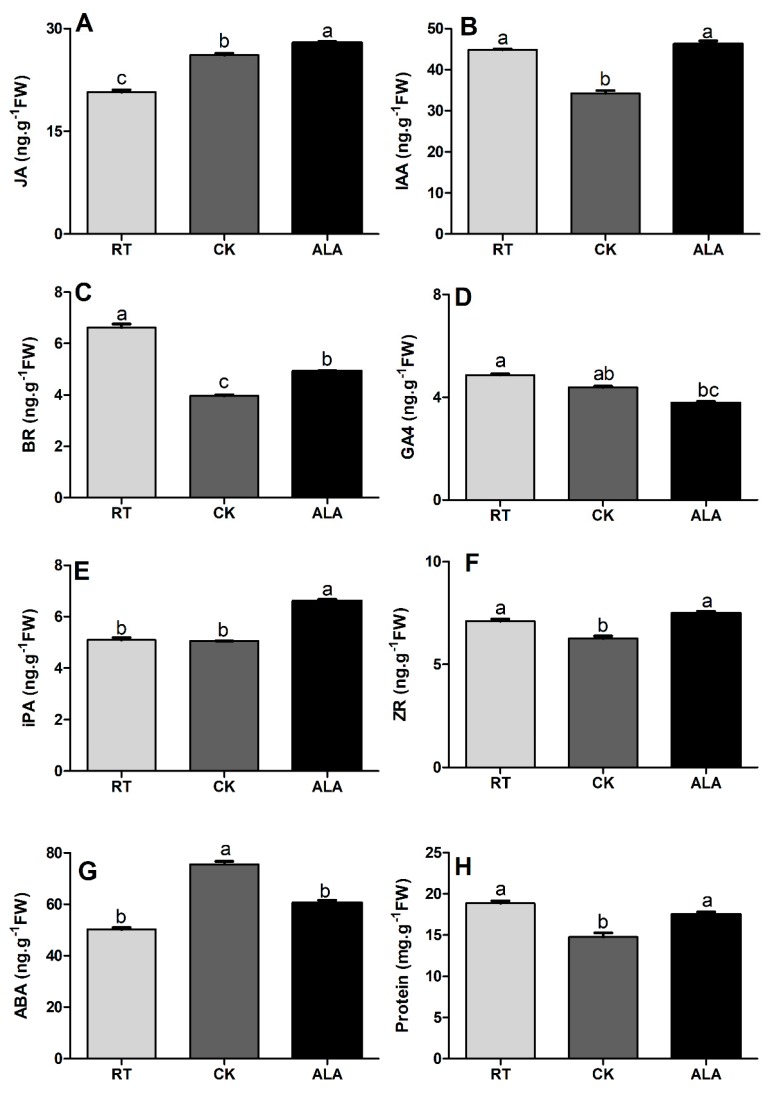
Effect of ALA on endogenous hormone accumulation under low-temperature stress conditions. **A**; Jasmonic Acid, **B**; Indole acetic acid, **C**; Brassinosteroid, **D**; Gibberellic Acid, **E**; Indole-3-propionic Acid, **F**; Zeatin riboside, **G**; Abscisic Acid, and **H**; Protein. RT= regular temperature; CK = control; ALA = 30 mg/L ALA. Different letters indicate a significant difference at *p* < 0.05.

Plant hormones play an important role in stress tolerance. Cucumbers subjected to low-temperature stress during the seedling stage had a significant reduction in endogenous hormone accumulation in their leaves (Figure 7). The result indicated that ALA significantly increased the levels of JA, IAA, BR, iPA, and zeatin-riboside (ZR) by 6.54%, 25.87%, 19.43%, 23.53%, and 16.34%, respectively, while GA4 and ABA decreased by 15.95% and 24.61%, respectively, compared to CK. Additionally, the RT treatment led to a significant increase in BR and GA4 contents when compared to the CK and ALA treatments, while IAA, ZR, and ABA were the same with ALA, but significantly higher than the CK treatment (Figure 7). The JA, iPA, and ABA contents in the RT treatment were significantly downregulated compared to CK and ALA. These results suggest that ALA induces endogenous hormones accumulation to increase low-temperature stress tolerance and enhanced growth of cucumber seedlings.

## 3. Discussion

ALA is a critical precursor in the tetrapyrrole biosynthetic pathway and is considered to be plant growth regulator that improves plant growth and stress tolerance [7,14]. Low-temperature stress can lead to the overproduction of ROS (H_2_O_2_ and O_2_^•−^), which are highly reactive, toxic, and as such have negative effects on chlorophyll content, photosynthetic rate, antioxidant enzyme activities, hormone, and nutrient accumulation [7,13,21]. Low-temperature stress inhibits melon and watermelon growth, chlorophyll levels, and photosynthetic capacity [21,22]. Our study shows that exogenous ALA application significantly reduces the damaging effects of low-temperature stress on cucumber seedlings, and leads to a significant increase in a number of plant growth parameters (plant height, root length, leaf area, dry weight, and strong seedling index) (Table 1). The results were similar to those of earlier studies, which reported that ALA alleviates the harmful effects of a range of environmental stresses (low temperature, salinity, and heavy metal stresses) by protecting chlorophyll and the photosynthetic machinery, stimulating a plant’s defensive response and increasing growth [22,23,24,25].

Chlorophyll (Chl) content is an important parameter frequently used to indicate chloroplast development [2,6,23]. Chl is sensitive to abiotic stresses and very easy to degrade [22], and can lead to a reduction in photosynthetic capacity [26,27]. Previous studies reported that low-temperature stress can induce a serious decline in chlorophyll content and photosynthetic capacity [22], leading to a significant reduction in plant growth [9,21,28]. ALA alleviate the harmful effects of salinity by regulating Chl synthesis pathway and leads to improve cucumber seedlings growth [14]. In the present study, chlorophyll content (Chl a, Chl b, Chl a+b, and carotenoids) significantly increased after ALA treatment (Figure 1). Additionally, ALA alleviated the degradation of chlorophyll and may be involved in chlorophyll biosynthesis or the inhibition of chlorophyll-degrading enzymatic activity [16,17,22]. Moreover, the results suggested that the decrease of Chl was lower in ALA-treated seedlings than CK, when compared to RT (Figure 1). The results are consistent with previous studies, which reported that ALA increased chlorophyll contents in tomato, cucumber, melon, and watermelon under drought, salinity and low-temperature stress [21,22,23,29]. The previous study reported that exogenous ALA application increases the activities of Glutamyl-tRNA reductase (GluTR) and glutamate-1-semiadelhyde 2,1-aminomutase (GSA-AT) enzymes, which catalyze the biosynthesis of ALA [30] and might induce the biosynthesis of Chl by inducing the expression of *psbA* and *psbD* under drought stress [31]. Transcriptome analysis indicated that ALA activated thousands of genes involved in a variety of biological process, e.g., Chl biosynthesis genes, the cell cycle, transcription factors, post-transcriptional regulation, and metabolism of macromolecules [16,23,32]. In addition, a previous study reported that ALA increased the expression levels of *ChlD*, *ChlH*, and *Chl1-1* genes, which are involved in Chl biosynthesis in cucumber, *B*. *napus* and Pakchoi [12,14,27]. These findings suggest that ALA protects Chl biosynthesis under low temperature, thus improving cucumber seedling growth (Table 1).

Photosynthesis is the basis of plant growth and development, and is sensitive to abiotic stresses [6,8,27,33]. Exogenous ALA application affects several physiological and biochemical processes, including photosynthesis whether under stress or normal conditions [34,35,36]. One recent study shows that the promotion of pepper seedling growth by exogenous ALA application under low-temperature stress leads to improvements in photosynthesis [37]. ALA is protecting photosynthetic machinery in numerus plant species from various kind of abiotic stresses and caused a significant increase growth [6,13,15,18]. The results of present study show that low-temperature stress induces a decline in photosynthetic capacity (*Pn*, *GS*, *Ci*, and *Tr*) in cucumber seedling leaves, but that capacity was significantly enhanced by exogenous ALA application (Figure 2). Previous studies have reported that exogenous ALA application increases photosynthetic capacity in cucumber and maize under chilling stress [9,38], Pakchoi under normal conditions [27], spinach under high-salinity and normal conditions [25], and oilseed rape under salinity and drought stress [8,15]. ALA might reduce the negative effects of low-temperature stress by increasing the photosynthetic capacity in cucumber seedlings (Figure 2). ALA is an essential precursor for chlorophyll biosynthesis under stress conditions [14,39,40], and may help increase harvest quantum under low-temperature stress [15,37,40]. The transcriptome analysis of Kentucky bluegrass suggested that ALA upregulates genes involved in photosynthesis, chloroplast developments, thylakoid membrane, and chlorophyll biosynthesis [30,31], which is the prime contributor of photosynthetic machinery [40,41,42,43]. Moreover, in the present study, ALA enhanced chlorophyll content (Figure 1), especially chlorophyll b, which might improve the ability of quantum harvesting of leaves leading to enhanced photosynthetic rate and probably improved growth [29,37,44].

To alleviate oxidative injury induced by stress, plants have evolved mechanisms to scavenge these toxic and reactive species through antioxidation of enzymatic and nonenzymatic systems, which leads to damage and possibly caused cell death [7,26,36,40,45]. SOD is a key antioxidant enzyme scavenger of O_2_^•−^, catalyzing the dismutation of superoxide radicals to H_2_O_2_ and O_2_, while CAT directly scavenges H_2_O_2_. APX and GR remove O_2_^•−^ and H_2_O_2_ by activating AsA and GSH (a nonenzymatic pathway) [7,33,40]. Our results show that ALA significantly increased antioxidant enzyme activities (SOD, POD, CAT, APX, and GR, Figure 3), while decreasing the MDA content (Figure 3F) and H_2_O_2_ and O_2_^•−^ contents in cucumber leaves under low-temperature stress (Figure 4). Previous studies reported that ALA-treated seedlings significantly increased antioxidant enzyme activities in cucumber under salinity and drought stress [19,29], pepper [37], and melon exposed to low-temperature stress [21]. In strawberry and rice seedlings, ALA activates a plant’s antioxidant defense system and the expression of defense-encoded genes (*SOD*, *POD*, *CAT*, and *APX*) to alleviate the damaging effects of salinity and photodynamic stresses [46,47], decreasing the overproduction of ROS and MDA [7,14,20,26,28]. The previous study suggested that CAT, POD, and APX contain a heme prosthetic group, while ALA is a key precursor of heme biosynthesis [18], which might be the reason that ALA-treated seedlings showed increased antioxidant enzyme activity (Figure 3), reducing the overproduction of ROS and MDA (Figure 4) in cucumber seedling under low-temperature stress. Thus, it can be concluded that exogenous ALA application increased low-temperature stress tolerance and stabilized ROS and MDA production, and might be associated with an increased expression of genes encoding antioxidant enzymes, like SOD, POD, CAT, APX, and GR, all of which resulted in a significant increase in cucumber seedling growth (Table 1) [24,26,29,48].

The uptake and distribution of essential nutrients are crucial for the maintenance of homeostasis and plant growth under unfavorable conditions [48,49]. Stress mostly limits nutrient (both macro and micro) acquisition and translocation in plant tissue [50,51]. Abiotic stress decreases ion homeostasis and accumulation in maize and halophyte, causing a significant reduction in plant growth, photosynthetic activity, and plant defenses [8,23,49]. Cucumber plants exposed to low-temperature stress had significantly decreased levels of vital macro- (N, P, K, Ca, and Mg) and micronutrients (Cu, Fe, Mn, and Zn) in the leaves and roots, but Mn increased in the roots (Figure 5 and Figure 6). Treatment with exogenous ALA significantly increased both macro- and micronutrient contents in cucumber seedling roots and leaves (Figure 5 and Figure 6). Earlier studies suggested that exogenous ALA application positively affected uptake, translocation, and accumulation of these essential nutrients in *Brassica napsus* [39] and watermelon [52]. ALA application also improved the uptake of essential nutrients under salinity stress in *Brassica napus* [8], while increasing nitrogen metabolism (NR, GOGAT enzymes activities) under NaCl stress in *Isatis indigotica* Fort and watermelon, leads to increased plant growth [52,53]. In this study, the nutrient contents in the ALA and RT treatments were almost similar, but both were significantly higher than CK (Figure 5 and Figure 6), which might be the reason that ALA leads to improve cucumber seedling growth. These results are consistent with previous studies, which reported that ALA regulates ion uptake under salinity and drought stress, and activates the defense system in *Brassica* spp. [8,41,52]. It can be concluded that ALA plays a key role in ion homeostasis and balance, which is involved in virtually all metabolic and cellular functions, such as energy metabolism, primary and secondary metabolism, gene and hormonal regulation, reproduction, and signal transduction pathways [6,20,38,47,54], thus improving growth (Table 1).

Plant resistance to abiotic stress is strongly associated with phytohormones that regulate various biological and developmental processes during growth [5,7,32,55]. Interaction and cross-talk between plant hormones can control a broad spectrum of physiological and developmental processes through the activation of various transcriptional factors [3,5,41]. The previous decade saw a rapid rise in studies of plant hormone signal transduction pathways and regulatory mechanisms [1,5,7,9,41]. The results of the present study suggest that exogenous ALA application induces endogenous hormone (BR, IAA, JA, SA, and iPA) to accumulate except ABA and GA4, (Figure 7). A recent study suggested that ALA increased ABA contents under drought stress in wheat seedlings, indicating that ALA interacts with endogenous plant hormones to induce stress tolerance [31]. The BR contents were significantly higher in the ALA treatment than CK (Figure 7). These are supported by a previous study, which reported that BR increases the transcriptional levels of the ethylene biosynthesis genes (*CsACS1*, *CsACS2*, *CsACS3*, *CsACO1*, *CsACO2*, and *CsAOX*) in response to abiotic stresses in cucumber, especially to low-temperature stress [56], while ryegrass and cucumber improved salt and low-temperature stress tolerance by increasing endogenous hormonal accumulation (BR, IAA, ABA, SA, JA, iPA, and GA4) and ion homeostasis (Na, K, Ca, and Mg), as treated with exogenous BR [1,55]. These findings strongly suggest that ALA interacts with endogenous phytohormones, especially JA, IAA, BR, iPA, and ZR, to induce low-temperature stress tolerance in cucumber. Taken together, ALA increases low-temperature stress tolerance by regulating endogenous hormone accumulation (Figure 7) to activate the plant’s defense system (Figure 3), protecting chlorophyll (Figure 1) and photosynthesis (Figure 2), improving cucumber seedling growth (Table 1). Recent studies reported that various phytohormones increased the plant defense system and reduced the overproduction of ROS, are support our findings [6,19,46,55].

Plant hormones and their cross-talk are important for plant growth and development [57], activating various transcriptional factors and signal transduction pathways, which play a fundamental role in plant defense system [55]. Cross-talk and interactions between ALA and phytohormones may have been implicated in the regulation of several developmental and physiological processes, including responses to abiotic stress, and for discovery of new genes and transcriptional factors.

This study provides insight into the hormonal regulation by exogenous ALA application to induce low-temperature stress tolerance. Low-temperature stress caused lipid peroxidation and decreased antioxidant enzyme activities, chlorophyll levels, photosynthetic capacity, and ion and hormone accumulation, thus inhibiting cucumber seedling growth. Exogenous ALA protects cucumber seedlings against low-temperature stress by regulating endogenous hormones; increasing Chl, photosynthetic capacity, and nutrient accumulation; antioxidant enzyme activities; and preventing lipid peroxidation. The application of 30 mg/L ALA could alleviate the harmful effects of low temperature by boosting the plant’s defense system and decreasing ROS production, thus enhancing low-temperature stress tolerance. ALA regulates chlorophyll accumulation and leads to a significant increase in photosynthetic capacity. In addition, ALA effectively increases endogenous hormone accumulation, which is a novel finding. The versatile role of ALA may be attributed to its interaction with plant hormones that activate the post-transcriptional factor of the target pathway to increase low-temperature stress tolerance in cucumber seedlings. More research is required to further elucidate the ALA mechanism and interaction with hormones that confer abiotic stress tolerance. 

## 4. Materials and Methods

### 4.1. Plant Material and Growth Condition

The experiment was conducted from March to November 2017 in a controlled growth chamber at the Institute of Vegetables and Flowers, Chinese Academy of Agricultural Sciences, Beijing, China. Cucumber *Cucumis sativus* L. Cv. Zhongnong 26, obtained from the Institute of Vegetables and Flowers, Chinese Academy of Agricultural Sciences was used. After germination on moist gauze in petri dishes in the dark at a 28 °C, the sprouting seeds were transplanted to a seedling tray (32-hole plate) filled with a soil medium and placed at 28/18 °C day/night temperature, with 70–75% humidity and 300–350 µmol·m^−2^·s^−1^ photosynetically-active radiation provided for 14 h. When cotyledons fully extended, same-size seedlings were transplanted to a plastic container (34 cm × 26 cm × 12 cm), 6 seedlings per container) filled with half-strength hoagland nutrient solution and allowed to grow for 7 days, then exposed to low-temperature stress.

The experiment consisted of two parts: in the 1st experiment we investigated the effect of different concentrations of ALA on cucumber seedling growth, and identified the best ALA level. In the 2nd experiment we explored the role ALA by using the selected treatment for further analysis; regulation of antioxidant enzymes activities, endogenous hormones, and nutrients accumulation.

### 4.2. Treatments and Sampling

Once fully expended, 1st-leaf-stage cucumber seedlings were divided into five groups as follows.

RT (Regular temperature; 28/18 °C day/night temperature)

CK (Control; 12/8 °C day/night temperature)

T1 (15 mg/L ALA + 12/8 °C day/night temperature)

T2 (30 mg/L ALA + 12/8 °C day/night temperature)

T3 (45 mg/L ALA + 12/8 °C day/night temperature)

ALA was sprayed on cucumber seedlings leaves until they were is wet, with three days’ interval. The CK treatments were treated with same concentration of ethanol, while RT was untreated (treated same amount of with water). ALA stock solution was prepared by dissolving ALA in ethanol and storing it at 4 °C, with 0.02% *v*/*v* Tween-20 was used as a surfactant. The whole experiment was repeated three times, with each treatment having three pots (containers). The treated seedlings were exposed to a low temperature at 12/8 °C day/night. The photoperiod was kept at 14 h. Seedlings were exposed to low-temperature stress for 7 days. The fully expended second and third leaves were sampled after 7 days, immediately snap-frozen in liquid nitrogen, and stored at −80 °C until required for analysis.

### 4.3. Measurement of Growth Parameters

Plant height, root length, and hypocotyl diameter were determined by using a ruler and digital Vernier calipers. To determine fresh weight, roots and shoots were separated and weighed, and the same plants were also used for leaf area determination. The same plants were placed in an oven at 105 °C for 30 min and then dried at 75 °C. These plants were weighed to record plant dry weight. The strong seedling index (SSI) was determined as follows.

(1) $$\;\mathrm{Strong}\;\mathrm{Seedling}\;\mathrm{Index}\;=(\frac{\mathrm{Hypocotyl}\;\mathrm{Diameter}}{\mathrm{Plant}\;\mathrm{Height}\;}+\;\frac{\mathrm{Root}\;\mathrm{Dry}\;\mathrm{Weight}\;}{\mathrm{Shoot}\;\mathrm{Dry}\;\mathrm{Weight}\;})\times \;\mathrm{Total}\;\mathrm{Dry}\;\mathrm{Weight}\;$$

### 4.4. Measurement of Chlorophyll Contents

Total chlorophyll contents were extracted in 95% ethanol. Chlorophyll contents were measured using a spectrophotometer [55].

### 4.5. Measurement of Gas Exchange Parameters

The net photosynthesis (*Pn*), stomatal conductance (*Gs*), transpiration rate (*Tr*), and intercellular CO_2_ concentration (*Ci*) on the second fully expended leaves were measured by using a portable photosynthesis system (LI-6400XT). Five plants with leaves of the same size were selected from each treatment under the controlled growth chamber between 11 am and 12 pm to ensure maximum photosynthesis [55].

### 4.6. Leaf Antioxidants Enzymes Activity and MDA Contents

Half a gram of fresh leaf was ground with a chilled pestle and mortar in 4 mL ice-cold 0.05 mol/L sodium phosphate buffer (pH 7.8). The homogenate was centrifuged at 10,500 rpm for 20 min at 4 °C. The supernatant was used to determined antioxidant activities. Superoxide dismutase activity (SOD) was determined by measuring its ability to inhibit the photochemical reduction of nitro blue tetrazolium (NBT) according to a previously described method [58,59]. The absorbance was read at 560 nm. Catalase (CAT) activity was measured as the decline in absorbance at 240 nm due to decrease of extinction of H_2_O_2_. Peroxide (POD) activity was measured as the increase in absorbance at 470 nm. Ascorbate peroxidase (APX) activity was measured by the increase in absorbance at 290 nm as ASA was oxidized. Glutathione reductase (GR) activity was measured depending on the rate of decrease in absorbance of NADPH at 340 nm [12,58]. The MDA content was determined by the method previously described [11,59].

### 4.7. Determination of H_2_O_2_ and O_2_^•−^ Contents

The concentration of H_2_O_2_ and O_2_^•−^ were determined by using an assay kit (COMINBIO) with a UV-1800 spectrophotometer, following the manufacturer’s instructions [60].

### 4.8. Total Nutrients Contents Determination

The total nutrient contents in plant root and leaf samples were determined by an element analyzer (Vario MAX CN Elemental Analyzer, Elementar, Hanau, Germany). The samples were first digested in HNO_3_ by using a microwave digestion system (Mars X press Microwave Digestion system, CEM, Matthews, NC, USA). Samples were then analyzed for total nutrient concentrations with an inductively coupled plasma optical emission spectrometer (ICP-OES, Optima 5300 DV, Perkin Elmer, Waltham, WA, USA). The Jaldal Method was used to determine total N content.

### 4.9. Leaf Hormones Extraction and Quantification

Leaf hormone content (ABA, IAA, GA4, JA, ZR, iPA, and EBR) was determined by ELISA (Enzyme Linked Immune Sorbent Assay) technology, as previously described [20]. The fresh samples (0.5 g leaf) were homogenized in liquid nitrogen and extracted in ice-cold methanol (80% *v*/*v*) with butylated hydroxytoluene (1 mmol/L) and kept at 4 °C overnight. The samples were centrifuged for 20 min at 10,000× *g* (4 °C), after which the extracts were passed through a C18 Sep-Pak Cartridge (water, Milford, MA, USA) and dried with liquid nitrogen. The residues were dissolved in PBS (0.01 mol/L, pH 7.4) to determine the hormone levels. Microtitration plates (Nunc) were coated with synthetic ABA, IAA, GA4, JA, ZR, iPA, and EBR ovalbumin conjugates in NaHCO_3_ buffer (50 mmol/L, pH 9.6) and kept at 37 °C overnight. Ovalbumin solution (10 mg/mL) was added to each well in order to block nonspecific binding. The samples were again incubated for 30 min at 37 °C, and then the desired hormones and antibodies were added and again incubated for 45 min at 37 °C. The antibodies against hormones were obtained as described by Zhao et al. [61]. Horseradish peroxidase-labeled goat antirabbit immunoglobulin was then added to each well and samples were again incubated for 1 h at 37 °C. The buffer enzyme substrate was added and the enzymatic reaction was carried out in the dark at 37 °C for 15 min. Reactions were stopped using 3 mol/L H_2_SO_4._ Finally, the absorbance was recorded at 490 nm. The hormone contents were calculated by adding a known amount of standard hormones to split extract [55].

### 4.10. Statistical Analysis

There were four independent biological replications for each treatment and the whole experiment was repeated three times. The data were analyzed using an analysis of variance (ANOVA), and treatments were compared using an LSD test (*p* < 0.05), performed with Statistix 8.1 software (Analytical Software, Tallahassee, FL, USA).

## Figures and Tables

**Table 1:** Effect of 5-aminolevulinic acid (ALA) on cucumber seedling growth under low-temperature stress.

No.	Hypocotyl (mm)	Root Length (cm)	Height (cm)	Leaf Area (cm^2^)	Total DW (g)	SSI
RT	5.01 ± 0.12 ^a^	17.50 ± 1.15 ^a^	7.50 ± 0.32 ^a^	97.21 ± 6.31 ^a^	0.74 ± 0.10 ^a^	0.57 ± 0.04 ^a^
CK	3.68 ± 0.06 ^c^	10.13 ± 0.85 ^c^	4.37 ± 0.25 ^c^	53.68 ± 2.83 ^c^	0.38 ± 0.03 ^d^	0.36 ± 0.03 ^c^
T1	3.76 ± 0.12 ^c^	10.88 ± 0.85 ^c^	4.63 ± 0.47 ^c^	50.42 ± 5.01 ^c^	0.44 ± 0.03 ^c^	0.41 ± 0.04 ^c^
T2	4.75 ± 0.23 ^b^	14.15 ± 1.07 ^b^	6.38 ± 0.25 ^b^	78.81 ± 4.51 ^b^	0.56 ± 0.03 ^b^	0.51 ± 0.05 ^b^
T3	4.72 ± 0.29 ^b^	14.25 ± 0.95 ^b^	6.13 ± 0.25 ^b^	73.56 ± 4.84 ^b^	0.53 ± 0.04 ^b^	0.49 ± 0.02 ^b^

RT; regular temperature; CK: control, T1: (15 mg/L ALA), T2: (30 mg/L ALA), T3: (45 mg/L ALA). Treatments indicated by the same letters are not significantly different at *p* < 0.05.
